# Supraglottic airway versus endotracheal intubation in out-of-hospital cardiac arrest: a systematic review and meta-analysis

**DOI:** 10.1016/j.resplu.2026.101491

**Published:** 2026-09-14

**Authors:** Bianca S. Costa, Maria Clara P. Colla, Jonathas W. de Morais, Luís Gustavo Rizzolli, Ellen de F. Rezende, Luigi P. Cenci, Iago T.C. Grillo, Louise W.L.C. Cruz, João Manoel Silva, Aleksandar R. Zivkovic, Tulio Caldonazo, Felipe S. Passos, Johannes Ehler, Ricardo Esper Treml

**Affiliations:** a Department of Medicine, Nove de Julho University, Bauru, Brazil; b Department of Medicine, University of Caxias do Sul, Caxias do Sul, Brazil; c Department of Medicine, Santo Amaro University, São Paulo, Brazil; d Department of Medicine, University of the Far South of Santa Catarina, Criciúma, Brazil; e Department of Medicine, University of Buenos Aires, Buenos Aires, Argentina; f Department of Medicine, Pontifical Catholic University of Paraná, Curitiba, Brazil; g Department of Postgraduate in Anesthesiology, Surgical Sciences and Perioperative Medicine, University of São Paulo, São Paulo, Brazil; h Department of Intensive Care Medicine, Hospital Israelita Albert Einstein, São Paulo, Brazil; i Department of Anesthesiology, Heidelberg University Medical Faculty, Heidelberg, Germany; j Department of Cardiothoracic Surgery, Jena University Hospital, Jena, Germany; k Department of Cardiothoracic Surgery, Weill Cornell Medicine, NY, USA; l Department of Thoracic Surgery, MaterDei Hospital, Salvador, Brazil; m Department of Anesthesiology and Intensive Care, Jena University Hospital, Jena, Germany; n Department of Anesthesiology, Perioperative and Pain Medicine, Stanford Medical School, CA, USA

**Keywords:** Supraglottic airway, Tracheal intubation, Out-of-hospital cardiac arrest, Survival, Return of spontaneous circulation, Favorable neurological outcome

## Abstract

**Purpose:**

Evidence comparing supraglottic airway with tracheal intubation in out-of-hospital cardiac arrest remains conflicting, and prior meta-analyses covered few randomized trials. We compared these strategies in adults.

**Methods:**

PubMed, Embase, and the Cochrane Library were searched from inception to January 2026 for randomized and observational studies comparing the two strategies in adults. Primary outcomes were survival to hospital discharge and return of spontaneous circulation (ROSC); favorable neurological outcome was secondary. Risk of bias was assessed with ROBINS-I and RoB 2, and certainty with GRADE. Risk ratios (RR) with 95% confidence intervals (CI) were pooled using restricted maximum likelihood random-effects models, with heterogeneity (*I*^2^), subgroup, sensitivity, meta-regression, and exploratory trial sequential analyses.

**Results:**

Thirteen studies (246,659 patients) were included. Survival to hospital discharge did not differ between SGA and TI groups (RR 0.96; 95% CI 0.82–1.12; *I*^2^ = 80%). Compared with tracheal intubation, supraglottic airway placement was associated with a lower rate of return of spontaneous circulation (RR 0.86; 95% CI 0.77–0.96; *I*^2^ = 96%), particularly in the prehospital setting (RR 0.82; 95% CI 0.71–0.95). The direction of the association differed according to study design: SGA was associated with a lower rate of ROSC than TI in observational studies (RR 0.81; 95% CI 0.73–0.89), whereas SGA was associated with a higher rate of ROSC than TI in the two randomized trials (RR 1.09; 95% CI 1.03–1.16). Favorable neurological outcome did not differ overall (RR 0.99; 95% CI 0.88–1.11; *I*^2^ = 61%), although the exploratory subgroup analysis showed a borderline statistically significantly lower rate of favorable neurological outcomes with SGA compared with TI among studies assessing outcomes at ≥1 month after the event (RR 0.90; 95% CI 0.81–0.99). Exploratory trial sequential analyses were overall inconclusive, with more supportive findings observed for ROSC. These exploratory analyses should be interpreted cautiously.

**Conclusion:**

In out-of-hospital cardiac arrest, survival to hospital discharge did not differ between strategies, whereas SGA placement, compared with TI, was associated with a lower likelihood of ROSC. In an exploratory subgroup analysis of studies reporting neurological outcomes at ≥1 month after OHCA, TI was associated with a higher likelihood of favorable neurological outcome compared with SGA**.** Given the mostly observational evidence, substantial heterogeneity, and reversal in randomized trials, these findings are hypothesis-generating and warrant randomized confirmation.

**Prospero Registration:** CRD420261337076.

## Graphical abstract

**Figure:**
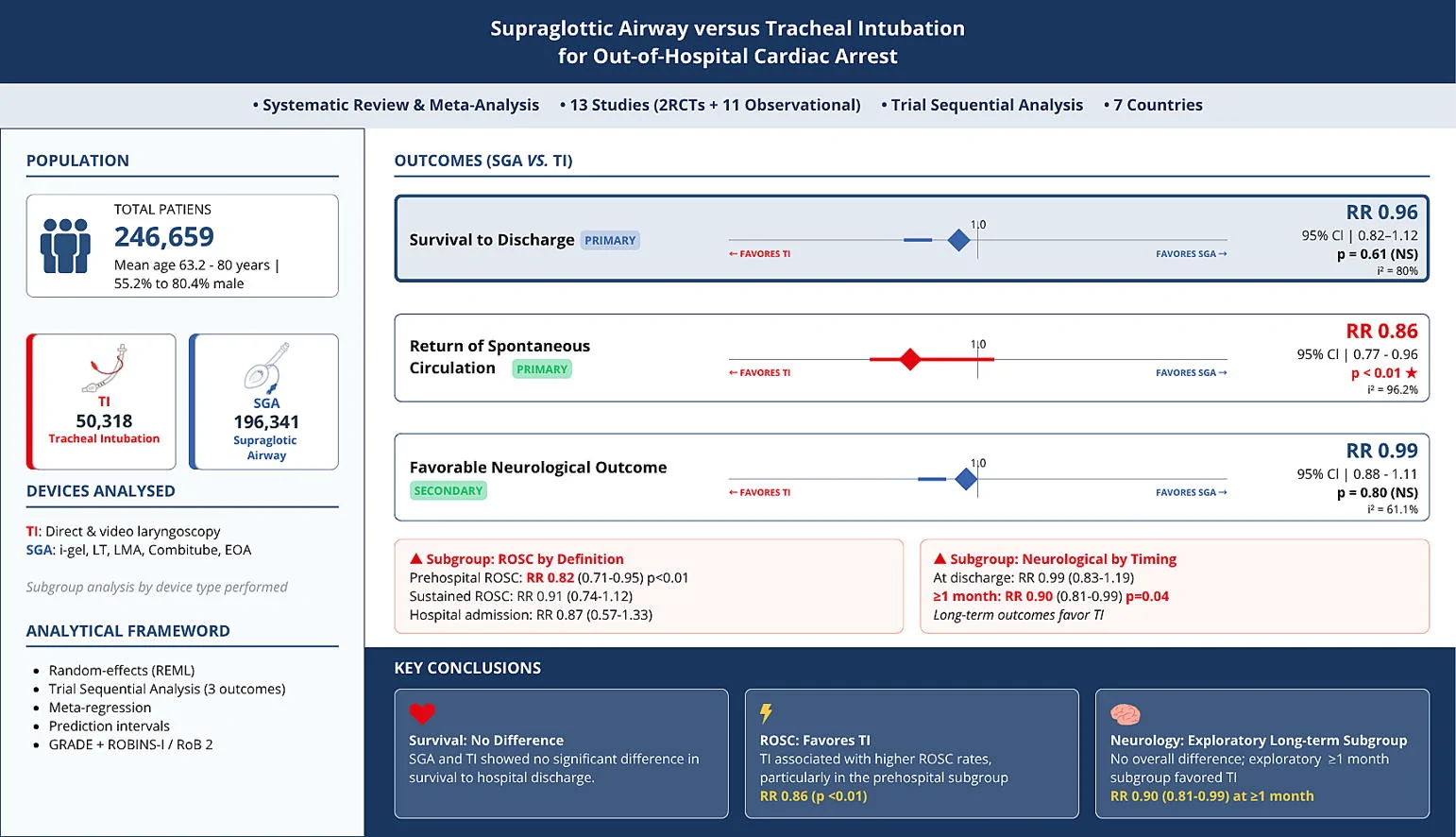

## Introduction

Out-of-hospital cardiac arrest (OHCA) affects approximately 81 per 100,000 person- years worldwide,^1^ with over 350,000 adults affected annually in the United States.^2^ Despite advances in resuscitation, survival remains below 10% in most registries.^2^, ^3^, ^4^ According to the guidelines, airway management is a key component of advanced cardiac life support and is commonly performed using tracheal intubation (TI) or supraglottic airway devices (SGAs).^4^, ^5^, ^6^

Tracheal intubation provides airway protection and effective ventilation and has been associated with improved survival to hospital admission.^7^, ^8^ However, prehospital TI carries well-documented risks: unrecognized esophageal intubation, prolonged laryngoscopy attempts, and interruptions in chest compressions that reduce coronary perfusion pressure.^9^, ^10^ In contrast, SGAs including the laryngeal mask airway (LMA), Esophageal-Tracheal Combitube, i-gel, and laryngeal tube (LT) are simpler, faster to insert, require less training, and facilitate broader use in prehospital settings, particularly in systems with limited TI capability.^11^, ^12^, ^13^ Like TI, SGAs are associated with aspiration, airway trauma, insertion failure, and device-related removal issues.^14^

Evidence comparing SGAs with TI remains controversial: some large cohort studies report comparable outcomes or superiority of SGAs,^15^, ^16^ whereas other studies and meta-analyses suggest TI may yield better results after cardiac arrest.^17^, ^18^ Previous meta-analyses have also yielded inconsistent findings.^18^, ^19^, ^20^ A recent meta-analysis conducted by Forestell et al. (2024)^20^ included randomized controlled trials (RCTs) and applied sequential trial analysis exclusively to survival outcomes. Furthermore, it included only four studies, which may have limited statistical power and subgroup exploration.

Therefore, we conducted a comprehensive, updated systematic review and meta-analysis, incorporating recently published large retrospective cohorts,^21^, ^22^, ^23^ to compare the impact of two airway strategies (SGA versus TI) on survival to hospital discharge, return of spontaneous circulation (ROSC), and favorable neurological outcome in adults with OHCA.

## Methods

This systematic review and meta-analysis were performed and reported in accordance with the Cochrane Collaboration Handbook for Systematic Review of Interventions^24^ and the Preferred Reporting Items for Systematic Reviews and Meta-analysis (PRISMA 2020) Statement guidelines.^25^ The study was registered in the International Prospective Register of Systematic Reviews (PROSPERO) under CRD420261337076 protocol.

### Search strategy

A literature search was conducted in PubMed, Embase, and the Cochrane Library on January 14, 2026, using predefined keywords related to OHCA, airway management, SGA, and TI. No language restrictions were applied. The complete search strategy is available in Supplementary Table 1.

### Study selection

Two independent reviewers (BSC and EFR) screened the records after removing duplicates. Discrepancies between reviewers were resolved through discussion and consensus, with a senior author (RET) making final decisions when necessary. Titles and abstracts were reviewed against predefined inclusion and exclusion criteria.

### Eligibility criteria

Studies were eligible if they met all of the following criteria: (I) RCTs or observational studies, including both prospective and retrospective designs; (II) cardiac arrest occurred out of hospital; (III) adult patients aged ≥18 years; (IV) a direct comparison between SGA and TI; and (V) reported at least one prespecified outcome of interest. We excluded animal studies, studies without a control group, and conference abstracts. More details are provided in Supplementary Table 2.

We excluded studies with overlapping populations, defined as cohorts derived from the same registry or overlapping datasets covering the same population and study period. In such cases, we excluded duplicate or overlapping datasets, and more recent studies or those with larger sample sizes and more complete data availability were preferentially included.

SGAs were defined according to the American Heart Association (2020)^26^ and prehospital evidence-based (2024)^11^ guidelines, which do not distinguish between device generations. Device classification is provided in Supplementary Table 3, and specific devices and intubation types are detailed in Table 1.

**Table 1:** Characteristics of included studies.

**Author, year**	**Study design**	**Country**	**Time frame**	**Groups**	**Sample**	**Type of SGA**	**Type of intubation**	**Risk of bias**
Battaglini, 2024*	Retrospective cohort	Italy	2017–2020	SGA TI	484 1218	NR	TI	Moderate
Behrens, 2020	Retrospective cohort	Germany	2007–2017	SGA TI	2776 2776	LT	TI	Moderate
Benger, 2018	RCT	England	2015–2017	SGA TI	4886 4410	I-gel	TI	Low
Cady, 2009	Retrospective cohort	United States	1997–2005	SGA TI	1487 4335	ETC	TI	Moderate
Chiang, 2018	Retrospective cohort	Taiwan	2008–2013	SGA TI	3099 1541	LMA	TI	Serious
Jung, 2022	Retrospective cohort	Korea	2015–2018	SGA TI	38,391 5592	NR	TI	Serious
Kang, 2016	Retrospective cohort	Korea	2010–2013	SGA TI	1634 1195	NR	TI	Moderate
Kim, 2025	Retrospective cohort	Korea	2019–2021	SGA TI	9379 1478	NR	TI-DL/TI-VL	Moderate
Lee, 2018	Retrospective cohort	Korea	2012–2014	SGA TI	160 162	I-gel	TI	Serious
McMullan, 2014	Retrospective cohort	United States	2011–2011	SGA TI	3110 5591	ETC/LMA/King tube	TI	Moderate
Tanabe, 2013	Retrospective cohort	Japan	2005–2007	SGA TI	122,194 16,054	LMA/EOA	TI	Serious
Wang, 2018	RCT	United States	2015–2017	SGA TI	1505 1499	LT	TI	Low
Yoshimura, 2024	Retrospective cohort	Japan	2014–2020	SGA TI	7236 4467	NR	TI	Moderate

*Abbreviations:* TI: Tracheal Intubation; TI-DL Tracheal Intubation with direct laryngoscope; TI-VL Tracheal Intubation with video laryngoscope; EOA: Esophageal Obturator Airway; ETC: Esophageal-tracheal combitube; LMA: Laryngeal mask airway; LT: Laryngeal tube; RCT: Randomized Controlled Trial; SGA: Supraglottic Airway; NR: Not Reported.

**^#^**Baseline characteristics were extracted from the propensity score–matched cohort, representing the population included in the adjusted analyses.

* Data derived from a post hoc analysis of the randomized controlled (TTM2 trial).

### Outcomes

The primary outcomes were survival to hospital discharge and return of spontaneous circulation (ROSC). The secondary outcome was favorable neurological outcome, defined according to the scales used in each study (e.g., CPC or mRS). Definitions of the secondary outcome in each study are summarized in Supplementary Table 4.

### Data extraction

Two reviewers (MCPC and LPC) independently extracted the data, and the accuracy of the data was verified by a third author (BSC). The extracted variables included study characteristics and patient demographics.

When propensity score matching (PSM) was applied, demographic and baseline characteristics were extracted from the matched cohorts. Conversely, for studies using inverse probability of treatment weighting (IPTW), baseline characteristics were obtained from the original unweighted cohorts, as weighting creates a pseudo-population for causal effect estimation.^27^

### Quality assessment and publication bias

Two independent authors (BSC and LGR) performed the assessments, and disagreements were resolved through discussion and consensus. The risk of bias of non-randomized studies was assessed using the Risk Of Bias In Non-randomized Studies of Interventions (ROBINS-I) tool^28^ while RCTs were assessed using the revised Cochrane risk-of-bias tool for randomized trials (RoB 2).^29^ For ROBINS-I, each study was judged as having low, moderate, serious, or critical risk of bias across seven domains. For RoB 2, each study was categorized as low risk, some concerns, or high risk of bias across five domains.

We explored the potential for publication bias by visual inspection of contour-enhanced funnel plots for the primary outcome. Additionally, if <10 studies were available, a secondary outcome with ≥10 studies was also assessed. Egger’s regression test was performed for outcomes with ≥10 studies, in accordance with Cochrane recommendations.^24^

### Assessment of certainty of evidence

The Grading of Recommendations, Assessment, Development, and Evaluation (GRADE) approach, specifically the GRADEpro Guideline Development Tool,^30^, ^31^ was applied to assess the certainty of evidence (high, moderate, low, or very low). The risk-of-bias domain was informed by the overall ROBINS-I and RoB 2 judgments, and certainty-of-evidence assessments were conducted after data extraction and finalized by consensus between the two reviewers.

Trial sequential analysis (TSA) was performed as a post hoc exploratory analysis to assess the risk of random errors and determine whether the available evidence was sufficiently powered. Although TSA was originally developed for meta-analyses of RCTs, it has also been applied to observational studies to assess whether current evidence is conclusive enough.^32^ For all outcomes with sufficient data, TSA was performed based on cumulative random-effects meta-analyses using the DerSimonian–Laird estimator. The required information size (RIS) was calculated assuming a two-sided *α* of 5%, *β* of 20% (power 80%), and a relative risk reduction of 20%, with the control event rate derived from the TI group.

Both conventional and trial sequential monitoring boundaries (under O’Brien–Fleming method) were generated for the SGA and TI groups. A cumulative Z-curve was constructed to assess the sufficiency and robustness of the available evidence relative to the boundaries. For the studies,^23^, ^33^ propensity score–matched data were used to reduce confounding in the adjusted analysis. Analyses were performed using TSA software version 0.9.5.10 Beta (The Copenhagen Trial Unit, Copenhagen, Denmark).

### Statistical analysis

Statistical analyses were restricted to outcomes reported in at least three studies. Outcomes that were assessed in fewer than three studies were not pooled quantitatively.

For binary outcomes, risk ratios (RRs) with 95% CIs were calculated; values <1 favored TI, and values >1 favored SGA. A restricted maximum likelihood (REML) random-effects model was applied to all outcomes to account for variability between studies. Statistical heterogeneity was assessed with the Cochrane Q test and the *I*^2^ statistic, which was classified as low (*I*^2^ < 25%), moderate (*I*^2^ 25–50%), or high (*I*^2^ > 50%).^34^ A *p*-value < 0.05 was considered to indicate statistical significance. To further characterize between-study variability, a 95% prediction interval (PI) was calculated under the REML model for meta-analyses including at least five studies, providing an estimate of the expected range of true effects in future comparable studies.^35^

We conducted exploratory subgroup analyses to explore clinical and methodological heterogeneity. For the primary outcomes, survival was stratified by SGA device type (reflecting differences in device mechanisms) and geographic region (Asian, European, and North American), given variations in EMS systems and prehospital care. ROSC was stratified according to the timing of outcome assessment because of heterogeneity in its definition and study design. The secondary outcome, favorable neurological outcome, was stratified by assessment timing.

Additionally, univariate meta-regression analyses were conducted using study-level covariates calculated as weighted averages based on the total sample size, including bystander CPR, non-shockable rhythms, witnessed arrest, age, and cardiac origin across the SGA and TI groups.

As an exploratory analysis, we performed separate meta-analyses using the adjusted odds ratios (ORs) reported by the original studies for the three outcomes of interest. In studies reporting adjusted ORs for more than two airway strategies, only one comparison was included to avoid double-counting participants. A cumulative random-effects meta-analysis was performed using the inverse-variance method with the restricted maximum-likelihood (REML) estimator.

To assess the robustness of the findings, a leave-one-out sensitivity analyses were performed for all outcomes. All statistical analyses were performed using R software.^36^

## Results

### Study selection

Fig. 1 presents the PRISMA 2020 flow diagram outlining the study selection process. A total of 752 records were identified through database searching of PubMed, Embase, and the Cochrane Library. After the removal of 272 duplicate records, 480 records underwent title and abstract screening. 64 reports were assessed for eligibility, and 13 studies met the inclusion criteria and were included in the final meta-analysis.^21^, ^22^, ^23^, ^33^, ^37^, ^38^, ^39^, ^40^, ^41^, ^42^, ^43^, ^44^, ^45^ These publications were released between 2009 and 2025. Most were observational cohorts, with only two RCTs. The studies originated from England, Germany, Italy, Japan, Korea, Taiwan, and the United States of America (USA). Table 1 summarizes the characteristics of the included studies, and the studies contributing to each outcome analysis are summarized in Supplementary Table 5.

**Fig. 1:**
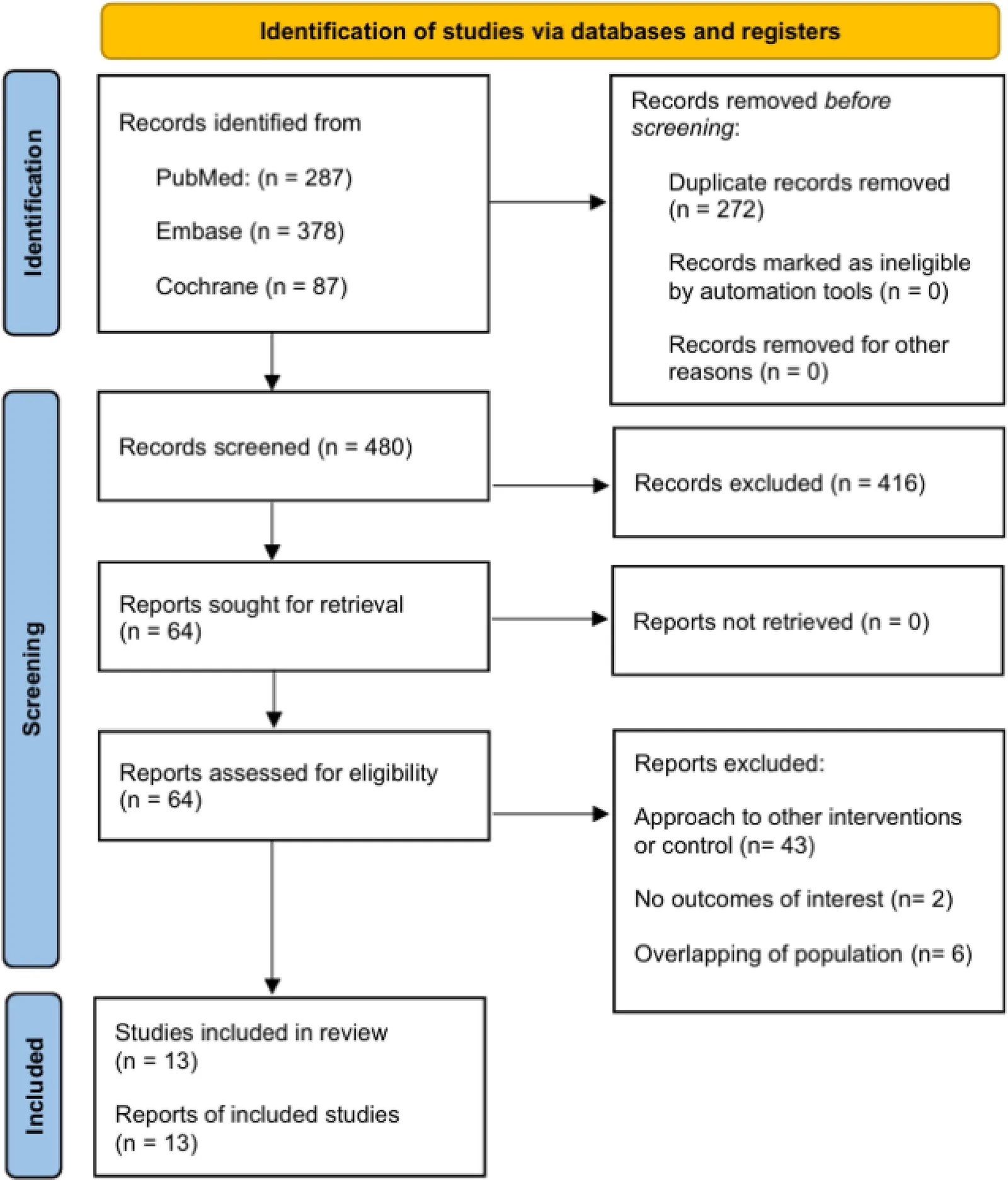
**Preferred Reporting Items for Systematic Reviews and Meta-Analyses (PRISMA) flow diagram of the study selection process**.

### Quality assessment and publication bias

The risk of bias for both observational studies (ROBINS-I tool) and RCTs (RoB 2 tool) is presented in Supplementary Figs. 1 and 2. Overall, most observational studies^33^, ^38^, ^41^, ^22^, ^23^ were classified as having moderate risk of bias, primarily due to potential confounding (including time-related bias) and selection of the reported results. Four studies were considered to have serious risk of bias, mainly due to confounding and deviations from intended interventions.^39^, ^40^, ^42^, ^44^ Regarding the two randomized clinical trials,^37^, ^45^ both were classified as having low risk of bias. These overall limitations are inherent to real-world evidence and should be considered when interpreting the pooled estimates.

The funnel plots did not demonstrate a high risk of publication bias based on visual inspection for either the survival or ROSC outcomes (Supplementary Fig. 3 and 4). To complement this subjective assessment, Egger’s regression test was performed for the ROSC outcome. The analysis indicated no evidence of publication bias (*t* = −0.60; df = 10; *p* = 0.5625).

### Assessment of certainty of evidence

The certainty of evidence was assessed for each outcome and stratified by study design (RCT vs observational studies). All outcomes from observational studies were rated as low certainty due to inherent limitations of observational design, susceptibility to uncontrolled bias, serious risk of bias in some studies (particularly in domain 1 of ROBINS-I), and substantial heterogeneity across outcomes.

In comparison, RCTs evidence ranged from very low to moderate certainty across outcomes, primarily limited by the small number of available trials. The favorable neurological outcome assessed by RCTs was rated as low certainty due to both the small number of studies and wide confidence intervals (Supplementary Table 6).

### Patient characteristics

Thirteen studies^21^, ^22^, ^23^, ^33^, ^37^, ^38^, ^39^, ^40^, ^41^, ^42^, ^43^, ^44^, ^45^ comprising a total of 246,659 patients with OHCA were included in the review. Of these, 196,341 individuals (81.1%) belong to the SGA group. The mean age ranged from 63.2 to 80 years, and the proportion of male patients varied between 55.2% and 80.4%. Most studies reported cardiac arrest of cardiac origin, with a proportion ranging from 33.1% to 100%, except for Yoshimura et al. (2024)^23^ study, which reported 0% of cardiac origin. Moreover, non-shockable rhythms were predominant, with the smallest study reporting 26.2%,^21^ and the proportion of witnessed cases spanned 7.6–91.3%. Supplementary Table 7 summarizes baseline characteristics of patients of the included studies.

In two cohorts,^23^, ^33^ the studies applied PSM, which enabled the extraction of patient characteristics and outcome data from the matched populations.

### Primary outcomes

#### Survival to hospital discharge

Pooled analysis of survival to hospital discharge, based on nine studies^22^, ^33^, ^43^, ^39^, ^40^, ^41^, ^42^ (Fig. 2), showed no significant difference between SGA and TI (RR 0.96; 95% CI 0.82–1.12; *p* = 0.61; *I*^2^ = 80%). The 95% prediction interval (0.58–1.58) suggests that future studies may show effects ranging from benefit to harm.

**Fig. 2:**
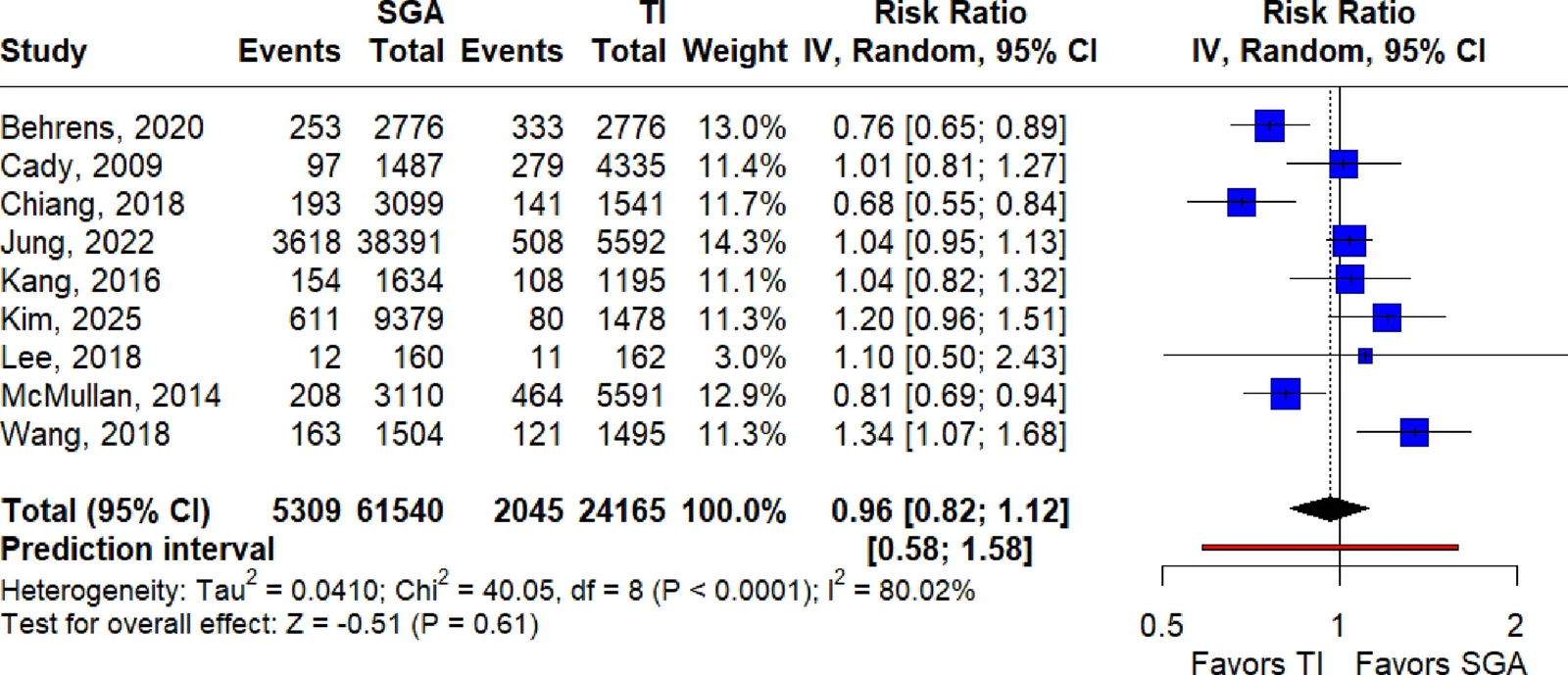
**Forest plot comparing survival to hospital discharge between supraglottic airway (SGA) and tracheal intubation (TI) in patients with out-of-hospital cardiac arrest (OHCA)**. CI: confidence interval; RR: risk ratio. **Note:** Outcome-specific denominators were extracted exactly as reported in the original publications. Consequently, studies such as Wang et al. contribute different denominators across outcomes because the original authors reported different numbers of patients with available data for each endpoint.

### Sensitivity analyses and subgroup analyses: survival to hospital discharge

Sequential leave-one-out sensitivity analysis confirmed that no single study materially altered the magnitude, direction, or statistical significance of the pooled estimate. Among all leave-one-out analyses, exclusion of Chiang et al. (2018)^39^ resulted in the greatest reduction in heterogeneity, from *I*^2^ = 80.0% to *I*^2^ = 75.8%; however, substantial heterogeneity remained (Supplementary Fig. 5).

In the subgroup analysis according to SGA device type, no significant differences in survival were observed for any subgroup. However, detailed device-level information was reported inconsistently across studies, limiting statistical power and the feasibility of exploratory subgroup analyses. The LT subgroup showed no significant difference in survival (RR 1.00; 95% CI 0.58–1.75; *p* = 0.99; *I*^2^ = 94%). The “other specified SGA” subgroup, which primarily included LMA, LT, Combitube, and esophageal obturator airway (EOA), also showed no significant difference in survival (RR 0.82; 95% CI 0.66–1.01; *p* = 0.07; *I*^2^ = 69.5%). LT was represented in this subgroup through the study by McMullan et al., which reported combined results for Esophageal-Tracheal Combitube, LMA, and LT.

Similarly, the “unspecified SGA” subgroup showed no significant difference (RR 1.06; 95% CI 0.98–1.14; *p* = 0.17; *I*^2^ = 0%). Finally, the i-gel subgroup, comprising a single study,^42^ also showed no significant difference in survival (RR 1.10; 95% CI 0.50–2.43). More information is provided in Supplementary Fig. 6.

In the second subgroup analysis, survival was evaluated according to geographic region. The single European study, compared with TI, SGA was associated with lower survival (RR 0.76; 95% CI 0.65–0.89), although this finding should be interpreted cautiously because it was derived from only one study.^33^ No statistically significant differences were observed in the North American (RR 1.02; 95% CI 0.76–1.37; *p* = 0.89; *I*^2^ = 85.1%) or Asian subgroups (RR 0.98; 95% CI 0.79–1.21; *p* = 0.84; *I*^2^ = 75.8%). Additional details are provided in Supplementary Fig. 7.

#### Trial sequential analysis: survival to hospital at discharge

The cumulative Z-curve did not cross either the conventional or trial sequential monitoring boundaries, and the accrued information represented only 1.47% of the RIS, indicating that, in our exploratory TSA including nine studies, the available evidence remains insufficient to draw definitive conclusions (Supplementary Fig. 8).

#### Exploratory analysis using adjusted ORs: survival to hospital discharge

As an exploratory analysis, a separate meta-analysis of adjusted ORs was performed for survival to hospital discharge using the five studies that reported adjusted estimates for the comparison of SGA versus TI (Supplementary Fig. 9). Studies included in the primary analysis were not eligible for this exploratory analysis if they did not report adjusted ORs for the comparison of interest or if the reported adjusted estimates used a different reference group. In the study by Kim et al., only one adjusted estimate was included to avoid double-counting participants; the comparison involving video laryngoscopy was selected because it included the larger sample size. The pooled analysis showed a borderline association favoring SGA (OR 1.24; 95% CI 1.00–1.54; *p* = 0.05; *I*^2^ = 53.5%). The 95% prediction interval (0.70–2.22) indicates that the true effect in future studies may range from no benefit to benefit, and these findings should be interpreted as exploratory.

### ROSC

Pooled analysis of ROSC^22^, ^23^, ^33^, ^37^, ^38^, ^39^, ^40^, ^41^, ^42^, ^43^, ^44^, ^45^ (Fig. 3) demonstrated that, compared with TI, patients managed with SGA had significantly lower ROSC rates (RR 0.86; 95% CI 0.77–0.96; *p* < 0.01; *I*^2^ = 96.17%; PI 0.56–1.31).

**Fig. 3:**
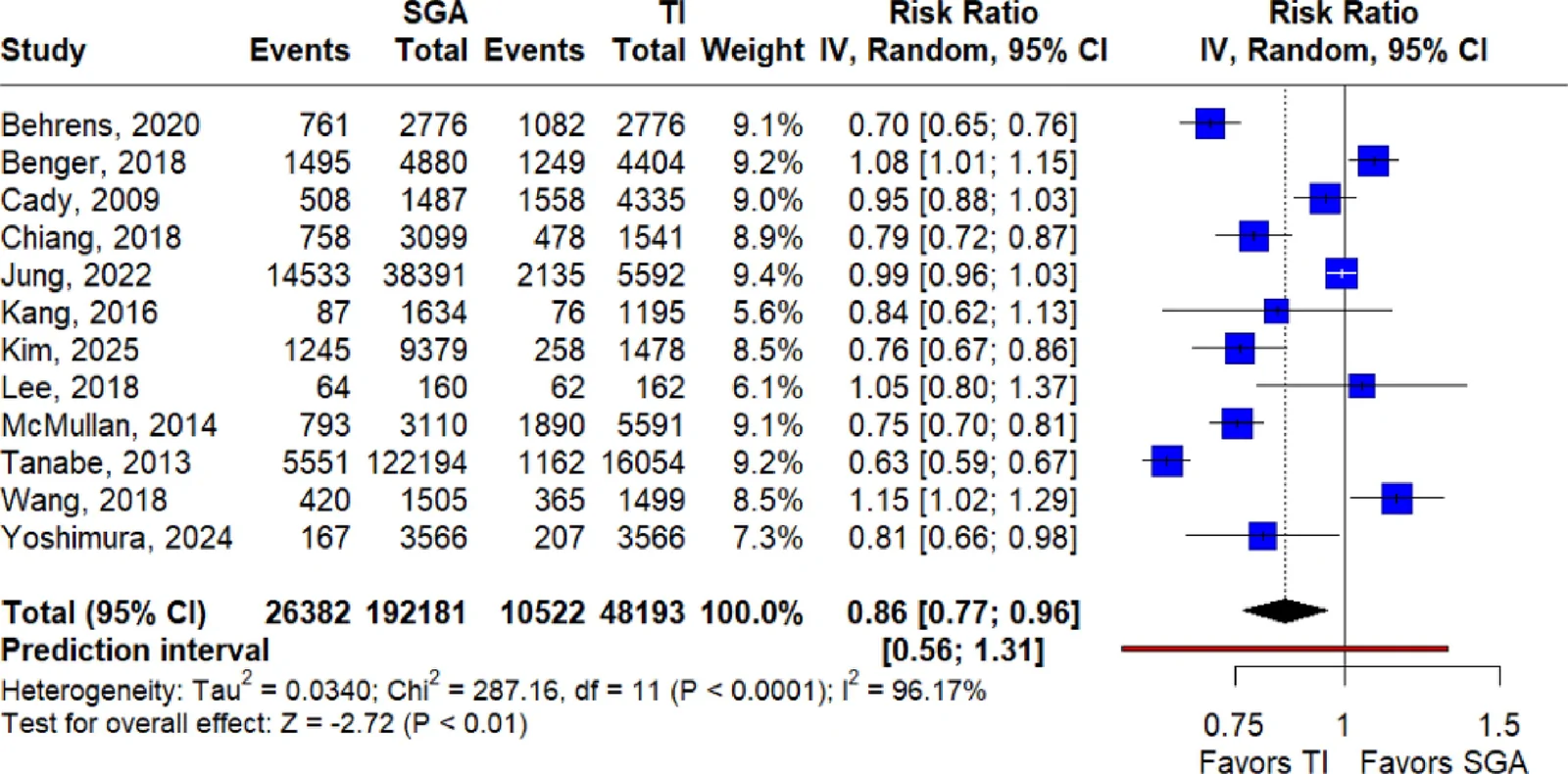
**Forest plot comparing return of spontaneous circulation (ROSC) between supraglottic airway (SGA) and tracheal intubation (TI) in patients with out-of-hospital cardiac arrest (OHCA)**. CI: confidence interval; RR: risk ratio. **Note:** Outcome-specific denominators were extracted exactly as reported in the original publications. Consequently, studies such as Benger et al. and Wang et al. contribute different denominators across outcomes because the original authors reported different numbers of patients with available data for each endpoint.

### Sensitivity analyses and subgroup analysis: ROSC

Leave-one-out sensitivity analyses showed that sequential exclusion of individual studies did not materially affect the pooled effect. Heterogeneity remained high, with no substantial reduction observed, which may affect interpretation (Supplementary Fig. 10).

Given that Yoshimura (2024)^23^ specifically examined OHCA due to drowning, its exclusion did not materially alter the overall ROSC estimate (RR 0.86; 95% CI 0.76–0.97; *p* < 0.01; *I*^2^ = 96.5%; Supplementary Fig. 10).

Exclusion of the high-weight study by Tanabe et al. (2013)^44^ did not materially change the ROSC estimate (RR 0.88; 95% CI 0.80–0.98; *p* = 0.02) or heterogeneity (*I*^2^ = 93.55%; Supplementary Fig. 10).

In the first subgroup analysis (Supplementary Fig. 11), according to different definitions of ROSC, no significant differences were observed for ROSC at hospital admission (RR 0.87; 95% CI 0.57–1.33; *p* = 0.52; *I*^2^ = 98.61%) or sustained ROSC (RR 0.91; 95% CI 0.74–1.12; *p* = 0.36; *I*^2^ = 92.2%). In contrast, SGA was associated with a significantly lower rate of prehospital ROSC compared with TI (RR 0.82; 95% CI 0.71–0.95; *p* < 0.01; *I*^2^ = 97.06%).

In the second subgroup analysis according to study design (Supplementary Fig. 12), SGA was associated with a significantly lower rate of ROSC compared with TI in observational studies (RR 0.81; 95% CI 0.73–0.89; *p* < 0.01; *I*^2^ = 95.83%). In contrast, among RCTs, SGA was associated with a significantly higher rate of ROSC compared with TI (RR 1.09; 95% CI 1.03–1.16; *p* < 0.01; *I*^2^ = 0%).

#### Meta-regression: ROSC

Meta-regression analyses showed no significant associations between ROSC and non-shockable rhythms (PEA and asystole) (*p* = 0.154), bystander CPR (*p* = 0.063), witnessed arrest (*p* = 0.141), age (*p* = 0.200), or cardiac origin (*p* = 0.186). Bubble plots for each covariate are presented in Supplementary Figs. 13–17. These variables were not associated with ROSC and did not account for the observed between-study heterogeneity.

### Trial sequential analysis: ROSC

In our exploratory TSA including 12 studies, the cumulative Z-curve crossed the conventional boundary after reaching the RIS, although it did not cross the trial sequential monitoring boundary. These findings suggest that the available evidence may be sufficient to support a statistically significant association for this outcome; however, this result should be interpreted cautiously given the predominance of observational studies and high heterogeneity, which may limit the reliability of the diversity-adjusted required information size and the boundary crossing (Supplementary Fig. 18).

#### Exploratory analysis using adjusted ORs: ROSC

Exploratory analysis using adjusted ORs (Supplementary Fig. 19) included eight studies, as fewer studies reported adjusted estimates than in the primary analysis. The pooled analysis suggested higher odds of ROSC with SGA compared with TI (OR 1.24; 95% CI 1.02–1.51; *p* = 0.03; *I*^2^ = 94.5%; PI = 0.63–2.44).

### Secondary outcome

#### Favorable neurological outcome

Pooled analysis of favorable neurological outcome^21^, ^22^, ^23^, ^33^, ^37^, ^39^, ^40^, ^41^, ^42^, ^43^, ^44^, ^45^ (Fig. 4) demonstrated no significant difference between SGA and TI (RR 0.99; 95% CI 0.88–1.11; *p* = 0.80; *I*^2^ = 61.1%; PI 0.69–1.41).

**Fig. 4:**
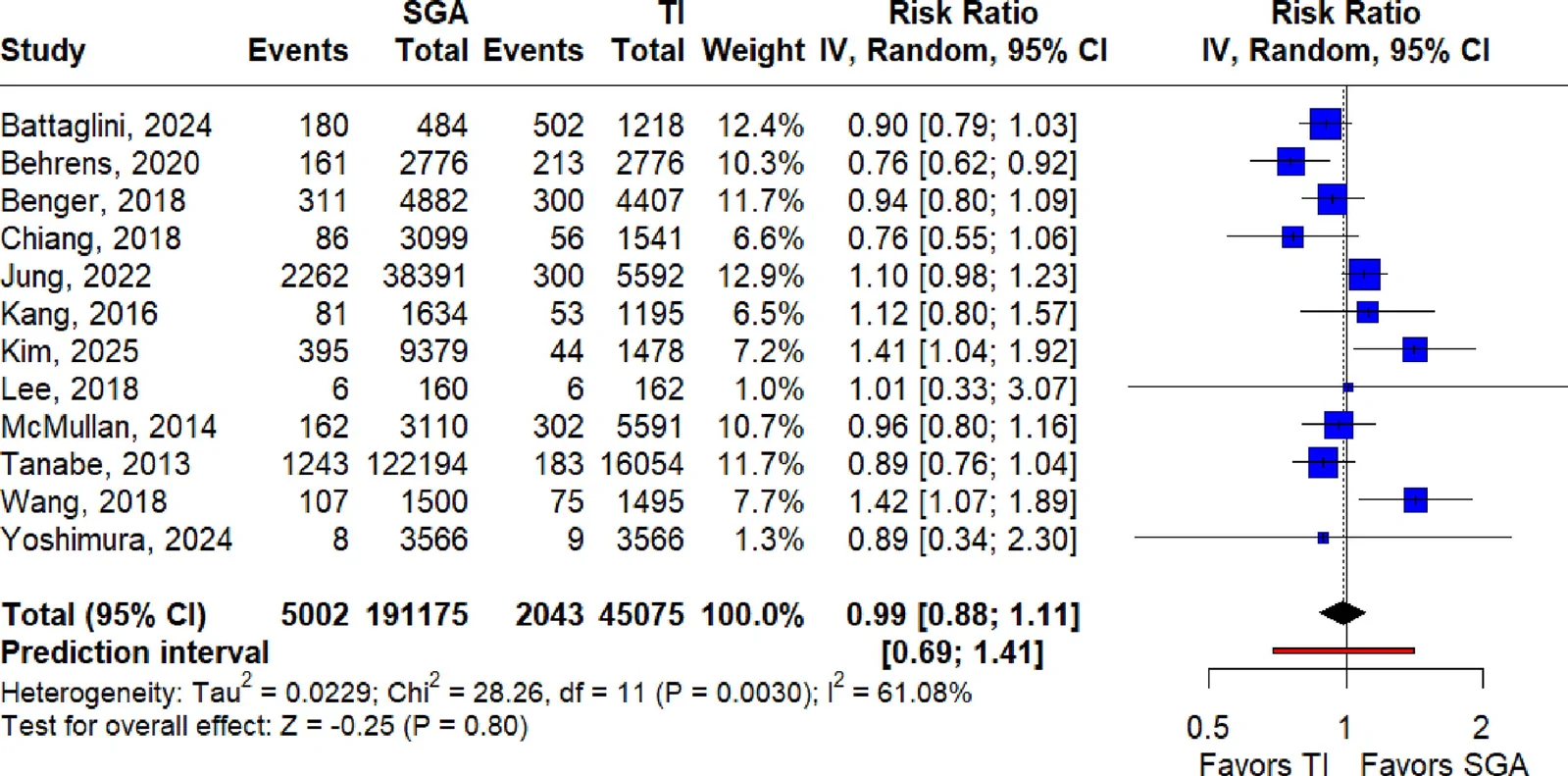
**Forest plot comparing favorable neurological outcome between supraglottic airway (SGA) and tracheal intubation (TI) in patients with out-of-hospital cardiac arrest (OHCA)**. CI: confidence interval; RR: risk ratio. **Note:** Outcome-specific denominators were extracted exactly as reported in the original publications. Consequently, studies such as Benger et al. and Wang et al. contribute different denominators across outcomes because the original authors reported different numbers of patients with available data for each endpoint.

#### Sensitivity analyses and subgroup analysis: favorable neurological outcome

Leave-one-out sensitivity analyses showed that sequential exclusion of individual studies did not materially affect the pooled effect estimate or its statistical significance. Exclusion of Wang et al. (2018)^45^ produced the greatest reduction in heterogeneity across all leave-one-out analyses, decreasing *I*^2^ from 61.1% to 53%; however, heterogeneity remained present (Supplementary Fig. 20).

In the subgroup analysis according to different definitions of favorable neurological outcome (Supplementary Table 3), no significant differences were observed for outcomes assessed at hospital discharge (RR 0.99; 95% CI 0.83–1.19; *p* = 0.94; *I*^2^ = 73.7%) or in studies where the timing of neurological assessment was not reported (RR 1.11; 95% CI 0.79–1.56; *p* = 0.55; *I*^2^ = 64.2%). In contrast, SGA was associated with a significantly lower rate of favorable neurological outcomes compared with TI in studies that evaluated neurological outcomes at ≥1 month after the event (OHCA) (RR 0.90; 95% CI 0.81–0.99; *p* = 0.04; *I*^2^ = 0%) (Supplementary Fig. 21).

#### Trial sequential analysis: favorable neurological outcome

In our exploratory TSA including 12 studies, the RIS was not rendered because the sample size of the first included study exceeded the calculated RIS. The cumulative Z-curve remained within the conventional boundaries after inclusion of all subsequent studies, indicating that no statistically significant treatment effect was demonstrated (Supplementary Fig. 22).

##### Exploratory analysis using adjusted ORs: favorable neurological outcome

Exploratory analysis using adjusted ORs (Supplementary Fig. 23) included seven studies, as fewer studies reported adjusted estimates than in the primary analysis. The pooled analysis showed no significant difference in favorable neurological outcome between SGA and TI (OR 1.11; 95% CI 0.86–1.42; *p* = 0.43; *I*^2^ = 74.2%; PI = 0.55–2.24).

## Discussion

In this systematic review and meta-analysis of thirteen studies including 246,659 patients, we comprehensively assessed the clinical implications of using SGA or TI in patients with OHCA. The main findings were as follows: (I) there were no significant differences in survival to discharge between the SGA and TI groups; (II) Compared with SGA, TI was associated with a higher likelihood of achieving ROSC; (III) in the subgroup analysis of prehospital ROSC, TI was associated with significantly higher ROSC compared with SGA; (IV) subgroup analyses according to study design demonstrated a significant ROSC advantage favoring TI in observational studies, whereas RCTs demonstrated a significant advantage favoring SGA; and (V) favorable neurological outcomes did not differ significantly between SGA and TI overall. However, in the exploratory subgroup analysis of studies evaluating neurological outcomes at ≥1 month after OHCA, TI was associated with a borderline statistically significant higher likelihood of favorable neurological outcome compared with SGA.

The higher prehospital ROSC observed with TI compared with SGA may have a plausible physiological basis. Tracheal intubation provides greater protection against aspiration and regurgitation by securing the airway directly in the trachea.^46^ Although TI requires greater technical proficiency and longer procedural time than SGA placement, successful intubation provides a more reliable airway, facilitating effective ventilation with minimal gas leakage and fewer interruptions in chest compressions.^47^, ^48^ Eickelmann et al. (2026)^47^ demonstrated significantly higher expiratory tidal volumes with TI than with SGA devices. Segond et al. (2025)^48^ demonstrated that supraglottic airway devices were associated with greater air leakage, which may compromise ventilation during CPR. Improved ventilation, together with high-quality chest compressions and minimal interruptions facilitated by a secured airway, may contribute to the higher ROSC observed with TI compared with SGA.^4^ However, our findings suggest that this potential benefit may be more relevant to prehospital ROSC than to ROSC overall.

An increased ROSC with a particular airway device, such as TI, does not necessarily translate into improved survival, which is consistent with our findings. ROSC reflects short-term hemodynamic gains achieved during resuscitation, whereas survival depends on additional post-arrest interventions. Key factors include targeted temperature management and early coronary reperfusion strategies.^49^, ^50^, ^51^ These findings highlight the importance of optimizing post-cardiac arrest management.

Our overall findings are consistent with the ROC Investigators’ registry analysis,^17^ which reported superior outcomes with TI compared with SGA, and with the meta-analysis by Benoit et al. (2015),^18^ which favored TI for ROSC. Although, the results from Forestell et al. (2024)^20^ found benefit for TI in ROSC supporting this study, it presented a lower heterogeneity. This finding may be explained not only by the smaller number of patients, but also by the inclusion of only RCts and the more homogeneous distribution of SGA types. In addition, these few studies found similar results favoring SGA, such as a pooled analysis showing 2.5 min faster time, and the lower variation in different types of supraglottic devices, with half of the studies using i-gel devices. In subgroup analyses stratified by study design, we found results consistent with the previous meta-analysis,^20^ showing that ROSC was favored by SGAs in RCTs, whereas observational studies favored TI. This divergence may reflect confounding by indication and resuscitation-time bias in observational studies, as TI is more often performed later or in patients with different prognoses, biases that are minimized in RCTs. Notably, the exploratory analyses using adjusted odds ratios reversed the direction of effect for ROSC, favoring SGA over TI; this suggests that residual confounding may partly drive the crude association favoring TI, and these adjusted results should be interpreted with caution.

Findings also differ from the PART trial,^45^ which reported higher 72-h survival with the laryngeal tube. This result may be explained by the higher initial success rate—almost 40% greater—with the use of LT, as well as differences in the timing of survival assessment compared to our study, which evaluated hospital discharge. While the PART trial was judged to have a low risk of bias, these device-specific characteristics may have influenced the observed treatment effect without compromising the methodological quality of the trial. These discrepancies likely reflect differences in included populations, SGA device types, and analytical approaches.

A more recent meta-analysis including 8 studies by Scholz et al. (2026)^52^ explored whether additional airway management training could influence device performance. The results suggested that such training may improve ROSC with SGAs, although no statistically significant difference between SGA and TI was observed. These findings underscore the need for further research, not only to directly compare SGA and TI, but also to evaluate how other factors may impact outcomes such as survival, ROSC, and favorable neurological outcomes.

Moreover, the considerable heterogeneity observed in our analysis may partly reflect structural differences among SGA devices (Supplementary Table 3). Greater heterogeneity was observed in subgroups including LT and mixed SGA categories (LMA, Combitube, and LT), which differ in airway sealing mechanisms and aspiration protection capabilities.^53^ These characteristics may have contributed to the variability in treatment effects observed across studies. However, these findings remain exploratory and should be interpreted with caution because of the small number of studies included in each subgroup.

The comparative effectiveness of TI and SGA is inextricably linked to provider skill and EMS system design. In physician-staffed systems, such as the German registry analyzed by Behrens et al. (2020),^33^ TI was associated with higher survival and better neurological outcomes, reflecting the higher first-pass intubation success rates achieved by experienced operators. In paramedic-based systems,^40^, ^45^ TI has also been associated with favorable outcomes compared with SGA when successfully performed, likely reflecting differences in provider expertise and EMS system characteristics rather than device effect alone.

These findings support current American Heart Association (2025)^4^ and European Resuscitation Council (2025)^46^ recommendations that TI should be the preferred airway strategy when performed by providers with adequate training and high first-pass success rates, given its association with higher ROSC compared with SGA and a favorable neurological outcome in the exploratory subgroup of studies reporting outcomes at ≥1 month. However, this latter neurological finding was borderline and should be interpreted cautiously. Gage et al. (2026)^16^ demonstrated that early transition to an alternative airway after a failed first intubation increases overall success by approximately 24% compared with continuing repeated intubation attempts. This supports the view that, when TI proficiency cannot be assured—as in many paramedic-based EMS systems—SGA devices remain a reasonable alternative, as survival to discharge did not differ between strategies. The wide prediction intervals observed across all outcomes mandate cautious interpretation, reinforce the need to individualize airway device choice based on provider skill, system resources, and patient factors, and indicate that future studies are needed to confirm these findings.

### Limitations

Several limitations should be acknowledged. Most included studies were observational, with only two employing propensity score matching, and residual confounding from severity driven airway selection and time-dependent resuscitation bias cannot be excluded, as reflected in the low to moderate GRADE certainty ratings. Heterogeneity was further introduced by inconsistent reporting of SGAs types, variation in outcome definitions and timing of neurological assessment, diverse emergency medical services systems and resuscitation protocols spanning 1997–2021 (study enrolment periods), and differences in provider training and post-resuscitation care. In particular, incomplete reporting of device-level SGA characteristics (including “Not Reported” in several large cohorts^21^, ^22^, ^23^, ^40^, ^41^) limited device-specific subgroup analyses.

Another limitation is the imbalance in patient numbers between SGA and TI groups, with SGA being used in approximately four times more patients. This reflects the predominance of Asian EMS systems among the included studies, which may influence generalizability and introduce confounding by indication related to airway selection.

Although funnel plots and Egger test suggested low risk of publication bias for return of spontaneous circulation, the limited number of studies for other outcomes constrained both bias assessment and meta regression analyses. Additionally, trial registries and gray literature were not systematically searched, and conference abstracts were excluded, which may have resulted in missed unpublished studies.

Trial sequential analysis findings should also be interpreted cautiously, given high between study heterogeneity and the limited number of randomized trials.

Future research should prioritize large pragmatic randomized controlled trials comparing second generation supraglottic airway devices with tracheal intubation, stratified by provider experience and system design, with standardized outcome definitions and long-term neurological outcome.

## Conclusion

In adults with OHCA, survival to hospital discharge did not differ significantly between strategies, whereas TI was associated with higher ROSC compared with SGA. An exploratory subgroup analysis of studies reporting neurological outcomes at ≥1 month after OHCA suggested a higher likelihood of favorable neurological outcome with TI compared with SGA; however, this finding was borderline and should be interpreted cautiously. These findings are hypothesis-generating and should be confirmed in prospective, device-stratified RCTs before informing clinical practice, reinforcing the need for individualized airway choices based on provider skill and the context of the emergency medical system.

## CRediT authorship contribution statement

**Bianca S. Costa:** Writing – original draft, Visualization, Supervision, Software, Project administration, Methodology, Investigation, Formal analysis, Data curation, Conceptualization. **Maria Clara P. Colla:** Visualization, Investigation, Data curation. **Jonathas W. de Morais:** Methodology, Data curation, Conceptualization. **Luís Gustavo Rizzolli:** Methodology, Formal analysis, Data curation. **Ellen de F. Rezende:** Investigation, Conceptualization. **Luigi P. Cenci:** Visualization, Data curation. **Iago T.C. Grillo:** Validation, Supervision. **Louise W.L.C. Cruz:** Writing – review & editing. **João Manoel Silva:** Visualization, Validation, Supervision. **Aleksandar R. Zivkovic:** Visualization, Validation, Supervision. **Tulio Caldonazo:** Writing – review & editing, Visualization, Validation, Supervision. **Felipe S. Passos:** Writing – review & editing, Visualization, Validation, Supervision. **Johannes Ehler:** Writing – review & editing, Validation, Supervision. **Ricardo Esper Treml:** Writing – review & editing, Writing – original draft, Visualization, Validation, Supervision, Methodology.

## Funding

TC was funded by the Deutsche Forschungsgemeinschaft (DFG, German Research Foundation) Clinician Scientist Program OrganAge funding number 413668513, by the Deutsche Herzstiftung (DHS, German Heart Foundation) funding number S/03/23 and by the Interdisciplinary Center of Clinical Research of the Medical Faculty Jena.

## Data availability statement

The data underlying this article are available in the article and in its online supplementary material.

## Declaration of competing interest

The authors declare that they have no known competing financial interests or personal relationships that could have appeared to influence the work reported in this paper.
