# Supplementary material for: Supraglottic airway versus endotracheal intubation in out-of-hospital cardiac arrest: a systematic review and meta-analysis
Source: Resusc Plus. 2026 Sep 14;32:101491. doi: 10.1016/j.resplu.2026.101491 (PMC13634401; doi:10.1016/j.resplu.2026.101491)
Supplement: Supplementary file 1 [file mmc1.docx]

***SUPPLEMENTARY MATERIAL***

**Supraglottic Airway versus Tracheal Intubation in Out-of-Hospital Cardiac Arrest: A Systematic Review and Meta-Analysis.**

# SUMMARY:

| **Supplementary Table 1.** Details of the Search Strategy | 4 |
| --- | --- |
| **Supplementary Table 2****.** Eligibility criteria. | 5 |
| **Supplementary Table 3.** Classification of supraglottic airway (SGA) devices according to generation, representative devices, and mechanism of action. | 6 |
| **Supplementary Table 4.** Definition of outcome of return of spontaneous circulation (ROSC) and favorable neurologic the included studies. | 7 |
| **Supplementary Table 5.** Studies contributing to each outcome analysis. | 8 |
| **Supplementary Table 6.** Summary of findings: Supraglottic airway devices versus tracheal intubation for adult patients with out-of-hospital cardiac arrest (GRADE Assessment). | 9-10 |
| **Supplementary Table 7.** Baseline characteristics of the included studies. | 11 |
| **Supplementary Figure 1.** Critical appraisal of studies according to Cochrane Collaboration’s Risk Of Bias In Non-randomized Studies of Interventions (ROBINS-I) tool. | 12 |
| **Supplementary Figure 2.** Critical appraisal of studies according to Cochrane Collaboration’s Risk Of Bias In Randomized Studies of Interventions (ROB2) tool. | 13 |
| **Supplementary Figure 3.** Assessment of publication bias for survival outcomes. | 14 |
| **Supplementary Figure 4.** Assessment of publication bias for ROSC outcomes. | 14 |
| **Supplementary Figure 5.** Leave-one-out sensitivity analysis (survival to discharge). | 15 |
| **Supplementary Figure 6.** Subgroup analysis according to different types of SGA (survival) | 15 |
| **Supplementary Figure 7.** Subgroup analysis according to Asian vs European/North American regions (survival). | 16 |
| **Supplementary Figure 8.** Trial Sequential Analysis (survival to discharge). | 17 |
| **Supplementary Figure 9.** Exploratory meta-analysis of adjusted odds ratios for survival to hospital discharge | 18 |
| **Supplementary Figure 10.** Leave-one-out sensitivity analyses (ROSC). | 18 |
| **Supplementary Figure 11.** Subgroup analysis according to different definitions of ROSC. | 19 |
| **Supplementary Figure 12.** Subgroup analysis according to study design (Observational and Randomized Clinical Trial) | 19 |
| **Supplementary Figure 13.** Bubble plot of the covariate non-shockable rhythms-PEA and asystole- (ROSC outcomes). | 20 |
| **Supplementary Figure 14.** Bubble plot of the covariate bystander CPR (ROSC outcomes). | 20 |
| **Supplementary Figure 15.** Bubble plot of the covariate witnessed arrest (ROSC outcomes). | 21 |
| **Supplementary Figure 16.** Bubble plot of the covariate age (ROSC outcomes). | 21 |
| **Supplementary Figure 17.** Bubble plot of the covariate cardiac origin (ROSC outcomes). | 22 |
| **Supplementary Figure 18.** Trial sequential analysis (ROSC). | 23 |
| **Supplementary Figure 19.** Exploratory meta-analysis of adjusted odds ratios ROSC comparing supraglottic airway versus tracheal intubation. | 24 |
| **Supplementary Figure 20.** Leave-one-out sensitivity analyses (Favorable Neurological Outcome). | 24 |
| **Supplementary Figure 21.** Subgroup analysis according to timing of neurological outcomes. | 25 |
| **Supplementary Figure 22.** Trial sequential analysis (favorable neurological outcome). | 26 |
| **Supplementary Figure 23.** Exploratory meta-analysis of adjusted odds ratios Favorable Neurological Outcome comparing supraglottic airway versus tracheal intubation. | 27 |

## Supplementary Table 1. Details of the Search Strategy

| **Search Strategy for Each Database** | |
| --- | --- |
| **PubMed** | ('out-of-hospital cardiac arrest' OR 'out of hospital cardiac arrest' OR ohca) AND ('advanced airway management' OR 'advanced airway device' OR 'supraglottic airway' OR 'supraglottic airway device' OR 'laryngeal mask airway' OR lma OR 'i-gel' OR 'laryngeal tube' OR 'combitube') AND ('tracheal intubation' OR 'endotracheal intubation' OR TI) |
| **Embase** | ('out-of-hospital cardiac arrest' OR 'out of hospital cardiac arrest' OR ohca) AND ('advanced airway management' OR 'advanced airway device' OR 'supraglottic airway' OR 'supraglottic airway device' OR 'laryngeal mask airway' OR 'lma' OR 'i-gel' OR 'laryngeal tube' OR 'combitube') AND ('tracheal intubation' OR 'endotracheal intubation' OR 'TI') |
| **Cochrane** | ('out-of-hospital cardiac arrest' OR 'out of hospital cardiac arrest' OR ohca) AND ('advanced airway management' OR 'advanced airway device' OR 'supraglottic airway' OR 'supraglottic airway device' OR 'laryngeal mask airway' OR lma OR 'i-gel' OR 'laryngeal tube' OR 'combitube' ) AND ('tracheal intubation' OR 'endotracheal intubation' OR TI) |

**Supplementary Table 2.** Eligibility criteria.

| **Domain** | **Inclusion Criteria** | **Exclusion Criteria** |
| --- | --- | --- |
| Study design | Randomized controlled trials (RCTs) and observational studies (prospective or retrospective) | Animal studies, conference abstracts |
| Population | Adults (≥18 years) with out-of-hospital cardiac arrest (OHCA) | Pediatric patients (<18 years) or in-hospital cardiac arrest |
| Intervention | Supraglottic airway (SGA) | Studies without an SGA group |
| Comparator | Tracheal intubation (TI) | Studies without an TI comparator or without a control group |
| Outcomes | Reported at least one prespecified outcome of interest | No outcome of interest reported |
| Population  Overlap | Largest or most complete dataset included when overlapping populations were identified | Duplicate or overlapping cohorts derived from the same registry or study population |

## Supplementary Table 3. Classification of supraglottic airway (SGA) devices according to generation, representative devices, and mechanism of action.

| **Generation** | **Main Characteristics** | **Representative Devices** | **Mechanism of Action** |
| --- | --- | --- | --- |
| First- generation SGA | Basic airway sealing without dedicated gastric drainage channel; lower sealing pressure | LMA Classic, LMA Unique, Flexible LMA, Cobra PLA, Air-Q, Esophageal-tracheal Combitube (ETC) | Ventilation occurs above the vocal cords using an inflatable cuff that seals the periglottic space. Devices do not include a gastric drainage channel; the Combitube uses a dual-cuff system allowing ventilation regardless of esophageal or tracheal placement while providing esophageal occlusion. |
| Second- generation SGA | Improved airway seal and safety profile with gastric drainage channel | LMA ProSeal, LMA Supreme, i-gel, Laryngeal Tube Suction (LTS), SLIPA, Ambu LMA | Enhanced pharyngeal sealing with higher airway pressures and gastric access for decompression, reducing aspiration risk and improving positive-pressure ventilation efficiency. |
| Third- generation SGA | Dynamic, self-adjusting sealing mechanism | Baska Mask | Non-inflatable self-sealing membrane adapts to airway anatomy, increasing seal pressure during ventilation and reducing air leakage. |

SGA devices were categorized according to generation based on structural design and airway sealing mechanisms as described in previous airway device reviews

(**El Naggar, A. M. S., Farid, D. M., Nasr, Y. M., & Alarga, E. N. O.** (2020). *Supraglottic Airway Devices: An Overview*. **Medical Journal of Cairo University**, **88**(1), 327–333. https://doi.org/10.21608/mjcu.2020.93994).

## Supplementary Table 4. Definition of outcome of return of spontaneous circulation (ROSC) and favorable neurologic the included studies.

| **Author and year** | **Definitions - ROSC** | **Definitions – Favorable neurologic** |
| --- | --- | --- |
| **Battaglini, 2024** | 20 min with signs of circulation without the need for chest compressions. | Defined as a modified Rankin Scale (mRS) score of 0–3 at 6 months after cardiac arrest.  6 months follow up |
| **Behrens, 2020** | Hospital admission with ROSC | Hospital discharge with cerebral performance category (CPC) 1 or 2. |
| **Benger, 2018** | Return of spontaneous circulation at arrival to the emergency department or hospital.   Hospital admission. | Modified Rankin Scale (mRS) at hospital discharge or 30 days, whichever occurred first.  Considered not reported in our subgroup analysis due to inclusion of two time periods. |
| **Cady, 2009** | Prehospital ROSC | Not Reported |
| **Chiang, 2018** | Sustained (2 hours) return of spontaneous circulation. | Cerebral Performance Category (CPC) 1–2 at discharge. |
| **Jung, 2022** | It does not define a minimum duration for ROSC.  Prehospital. | Survival to hospital discharge defined as CPC 1 or 2. |
| **Kang, 2016** | It does not define a minimum duration for ROSC.  Prehospital. | Survival to hospital discharge defined as CPC 1 or 2. |
| **Kim, 2025** | Any prehospital ROSC. | Cerebral Performance Category (CPC) 1 or 2. Time Not Reported. |
| **Lee, 2018** | Was defined as maintaining spontaneous circulation for more than 20 minutes.  Sustained ROSC | Cerebral Performance Category (CPC) 1 or 2. Time not reported. |
| **McMullan, 2014** | Restoration of pulses with circulation present for at least 20 minutes.  Sustained ROSC. | Cerebral Performance Category (CPC) 1 or 2. Hospital discharge. |
| **Tanabe, 2013** | Return of spontaneous circulation before hospital arrival.  Prehospital ROSC. | Cerebral Performance Category (CPC) 1 or 2 at 1-month survival. |
| **Wang, 2018** | Presence of palpable pulses on emergency department arrival.  Hospital admission ROSC. | Modified Rankin Scale (mRS) ≤3 at hospital discharge. |
| **Yoshimura, 2024** | Any ROSC reported in the registry.  Pre-hospital care or upon arrival at the hospital. | Cerebral Performance Category (CPC) 1 or 2 at 1-month survival. |

## Supplementary Table 5. Studies contributing to each outcome analysis.

| **Author and year** | **Survival To Hospital Discharge** | **Return of Spontaneous Circulation (ROSC)** | **Favorable Neurological Outcome** |
| --- | --- | --- | --- |
| Battaglini, 2024 |  |  | X |
| Behrens, 2020 | X | X | X |
| Benger, 2018 |  | X | X |
| Cady, 2009 | X | X |  |
| Chiang, 2018 | X | X | X |
| Jung, 2022 | X | X | X |
| Kang, 2016 | X | X | X |
| Kim, 2025 | X | X | X |
| Lee, 2018 | X | X | X |
| McMullan, 2014 | X | X | X |
| Tanabe, 2013 |  | X | X |
| Wang, 2018 | X | X | X |
| Yoshimura, 2024 |  | X | X |

## Supplementary Table 6. Summary of findings: Supraglottic airway devices versus tracheal intubation for adult patients with out-of-hospital cardiac arrest (GRADE Assessment).

| **SGA compared to TI for Adult patients with out-of-hospital cardiac arrest (OHCA).** | | | | | |
| --- | --- | --- | --- | --- | --- |
| **Patient or population:** Adult patients with out-of-hospital cardiac arrest (OHCA).  **Setting:**  **Intervention:** SGA  **Comparison:** TI | | | | | |
| **Outcomes** | **№ of participants  (studies)  Follow-up** | **Certainty of the evidence  (GRADE)** | **Relative effect  (95% CI)** | **Anticipated absolute effects** | |
|  |  |  |  | **Risk with TI** | **Risk difference with SGA** |
| Survival - Observational  assessed with: RR | 82706  (8 non-randomised studies) | ⨁⨁◯◯  Low^a,b^ | **RR 0.92**  (0.79 to 1.06) | 85 per 1.000 | **7 fewer per 1.000**  (from 18 fewer to 5 more) |
| Survival - RCT  assessed with: RR | 2999  (1 RCT) | ⨁◯◯◯  Very low^c,d^ | **RR 1.34**  (0.79 to 1.06) | 81 per 1.000 | **28 more per 1.000**  (from 17 fewer to 5 more) |
| ROSC- Observational  assessed with: RR | 228086  (10 non-randomised studies) | ⨁⨁◯◯  Low^a,e^ | **RR 0.81**  (0.73 to 0.89) | 211 per 1.000 | **40 fewer per 1.000**  (from 57 fewer to 23 fewer) |
| ROSC- RCT  assessed with: RR | 12288  (2 RCTs) | ⨁⨁⨁◯  Moderate^c^ | **RR 1.09**  (1.03 to 1.16) | 273 per 1.000 | **25 more per 1.000**  (from 8 more to 44 more) |
| Favorable Neurological - Observational  assessed with: RR | 223966  (10 non-randomised studies) | ⨁⨁◯◯  Low^a,f^ | **RR 0.96**  (0.85 to 1.08) | 43 per 1.000 | **2 fewer per 1.000**  (from 6 fewer to 3 more) |
| Favorable Neurological - RCT  assessed with: RR | 12284  (2 RCTs) | ⨁⨁◯◯  Low^c,g^ | **RR 1.13**  (0.75 to 1.70) | 64 per 1.000 | **8 more per 1.000**  (from 16 fewer to 44 more) |
| ***The risk in the intervention group** (and its 95% confidence interval) is based on the assumed risk in the comparison group and the **relative effect** of the intervention (and its 95% CI).   **CI:** confidence interval; **RR:** risk ratio | | | | | |
| **GRADE Working Group grades of evidence**  **High certainty:** we are very confident that the true effect lies close to that of the estimate of the effect.  **Moderate certainty:** we are moderately confident in the effect estimate: the true effect is likely to be close to the estimate of the effect, but there is a possibility that it is substantially different.  **Low certainty:** our confidence in the effect estimate is limited: the true effect may be substantially different from the estimate of the effect.  **Very low certainty:** we have very little confidence in the effect estimate: the true effect is likely to be substantially different from the estimate of effect. | | | | | |

**Explanations**

a. Most studies were observational, and four studies presented bias mainly in domain 1 (D1) of ROBINS-I.

b. Substantial heterogeneity was observed for this outcome (I² > 80%).

c. Although all included studies were randomized trials, only a few studies were available.

d. Only one study contributed to this analysis.

e. Substantial heterogeneity was observed for this outcome (I²> 95%)

f. Substantial heterogeneity was observed for this outcome (I²>55%)

g. Confidence interval is wide and crosses no effect, indicating imprecision

## Supplementary Table 7. Baseline characteristics of the included studies.

| **Author, year** | **Groups** | **Sample** | **Mean age** | **Male, n(%)** | **PEA/ASY, n(%)** | **Witnessed, n(%)** | **Cardiac origin, n(%)** | **Bystandard CPR** |
| --- | --- | --- | --- | --- | --- | --- | --- | --- |
| Battaglini, 2024 | SGA  TI | 484  1,218 | 65.2  63.2 | 368 (76.0)  979 (80.4) | 144 (29.8)  319 (26.2) | 442 (91.3)  1,110 (91.1) | 484 (100*)  1,218 (100*) | 365 (75.4)  993 (81.5) |
| Behrens, 2020 | SGA  TI | 2,776  2,776 | 70.9  70.4 | 1862 (67.1)  1862 (67.1) | 2,213 (77.5)  2,424 (77.5) | 1,236 (44.5)  1,236 (44.5) | 2,262 (81.5)  2,262 (81.5) | 940 (33.9)  940 (33.9) |
| Benger, 2018 | SGA  TI | 4,886  4,410 | 73.0^@^  74.0^@^ | 3,132 (64.1)  2,791 (63.3) | 3,658 (76.3)  3,293 (76.3) | 3,101 (63.5)  2,788 (63.3) | 4,886 (100*)  4,410 (100*) | 3,149 (64.5)  2,774 (63.0) |
| Cady, 2009 | SGA  TI | 1,487  4,335 | 64  67 | 900 (60.6)  2,639 (60.9) | 1,065 (71.6)  3,204 (73.9) | 654 (44.0)  2,222 (51.28) | 1,487 (100)  4,335 (100) | 147 (9.9)  397 (9.2) |
| Chiang, 2018 | SGA  TI | 3,099  1,541 | 78.0^@^  78.0^@^ | 1,968 (63.5)  961 (62.4) | 2,152 (77.5)  2,152 (77.5) | 1,051 (33.9)  504 (32.7) | 3,099 (100*)  1,541 (100*) | 936 (30.2)  463 (30.1) |
| Jung, 2022 | SGA  TI | 38,391  5,592 | NR | 24,784 (64.6)  3,606 (64.5) | 31,040 (80.9)  4,528 (81.0) | 19,903 (51.8)  2,910 (52.0) | 38,391 (100*)  5,592 (100*) | 9,124 (23.8)  1,372 (24.5) |
| Kang, 2016 | SGA  TI | 1,634  1,195 | 68.0^@^  70.0^@^ | 1,116 (68.3)  769 (64.4) | 1,427 (87.3)  1,082 (90.5) | 845 (51.7)  643 (53.8) | 1,634 (100*)  1,195 (100*) | 243 (14.9)  116 (9.7) |
| Kim, 2025 | SGA  TI | 9,379  1,478 | 75.0^@^  74.0^$^ | 5,975 (63.7)  946 (64.1) | 8,008 (85.4)  1,283 (86.8) | 4,138 (44.1)  726 (49.6) | 9,379 (100*)  1,478  (100*) | 6,021 (64.2)  950 (64.3) |
| Lee, 2018 | SGA  TI | 160  162 | 65.65  66.15 | 109 (68.1)  96 (59.3) | NR | 84 (52.5)  90 (55.6) | 53 (33.1)  55 (34.0) | 50 (31.3)  64 (39.5) |
| McMullan, 2014 | SGA  TI | 3,110  5,591 | 63.9  66.1 | 1,934 (62.2)  3,382 (60.5) | 2,407 (77.4)  4,438 (77.6) | 1,490 (47.9)  2,689 (48.1) | 3,110 (100*)  5,591 (100*) | 1,101 (35.4)  2,113 (37.8) |
| Tanabe, 2013 | SGA  TI | 122,194  16,054 | 73.2  73.8 | 73,677 (60.3)  9,397 (58.5) | 109,267 (89.4)  14,517 (90.4) | 48,315 (39.5)  7,126 (44.4) | 71,854 (58.9)  8,594 (53,5) | 47,786 (39.1)  6,722 (41.9) |
| Wang, 2018 | SGA  TI | 1,505  1,499 | 64.0^@^  64.0^@^ | 928 (61.7)  901 (60.1) | 1,160 (77.1)  1,197 (79.9) | 691 (51.0)  708 (50.6) | 1,505 (100*)  1,499 (100*) | 698 (55.5)  709 (55.4) |
| Yoshimura, 2014 | SGA  TI | 7,236  4,467 | 80.0^@^  80.0^@^ | 4,016 (55.5)  2,375 (53.2) | 7,133 (98.5)  4,379 (98.0) | 533 (7.33)  372 (8.34) | 0 (0)  0 (0) | 3,657 (50.5)  2,267 (50.7) |

**Abbreviations**: ASY: Asystole; CPR: Cardiopulmonary Resuscitation; TI: Tracheal Intubation; PEA: Pulseless Electrical Activity; SGA: Supraglottic Airway; NR: Not Reported.

* Data derived from a post hoc analysis of the randomized controlled (TTM2 trial).

^@^ Age is reported as median.

\# Baseline characteristics were extracted from the propensity score–matched cohort, representing the population included in the adjusted analyses.

^$^This value represents a weighted mean derived from the two TI groups reported in the study. Median values were converted to means using the method described by Luo et al. (2018) (<https://www.math.hkbu.edu.hk/~tongt/papers/median2mean.html>).

**Supplementary Figure 1**. Critical appraisal of studies according to Cochrane Collaboration’s Risk Of Bias In Non-randomized Studies of Interventions (ROBINS-I) tool.

## Supplementary Figure 2. Critical appraisal of studies according to Cochrane Collaboration’s Risk Of Bias In Randomized Studies of Interventions (ROB2) tool.

## Supplementary Figure 3. Assessment of publication bias for survival outcomes.

## Supplementary Figure 4. Assessment of publication bias for ROSC outcomes.

## Supplementary Figure 5. Leave-one-out sensitivity analysis (survival to discharge). RR: risk relative; CI: Confidence Interval; PI: Prediction Interval.

## Supplementary Figure 6. Subgroup analysis according to different types of SGA (survival).CI: Confidence Interval. “Other specified SGAs” were defined as studies including different types of SGA devices with distinct designs and mechanisms, such as the Combitube, laryngeal tube, and laryngeal mask airway, representing a heterogeneous group comprising devices from different generations. “Unspecified SGA” included studies that did not report which type of SGA was used.

## Supplementary Figure 7. Subgroup analysis according to Asian, European and North American regions (survival). CI: Confidence Interval.

## Supplementary Figure 8. Trial sequential analysis for survival to hospital discharge

## Supplementary Figure 9. Exploratory meta-analysis of adjusted odds ratios for survival to hospital discharge comparing supraglottic airway (SGA) versus tracheal intubation (TI). CI = Confidence Interval

## Supplementary Figure 10. Leave-one-out sensitivity analyses (ROSC). RR: risk ratio; CI: Confidence Interval; PI: Prediction Interval.

## Supplementary Figure 11. Subgroup analysis according to different definitions of ROSC. CI: Confidence Interval.

## Supplementary Figure 12. Subgroup analysis according to study design (Observational and Randomized Clinical Trial)

## Supplementary Figure 13. Bubble plot of the covariate non-shockable rhythms-PEA and asystole- (ROSC outcomes).

## Supplementary Figure 14. Bubble plot of the covariate bystander CPR (ROSC outcomes).

## Supplementary Figure 15. Bubble plot of the covariate witnessed arrest (ROSC outcomes).

## Supplementary Figure 16. Bubble plot of the covariate age (ROSC outcomes).

## Supplementary Figure 17. Bubble plot of the covariate cardiac origin (ROSC outcomes).

## Supplementary Figure 18. Trial sequential analysis for return of spontaneous circulation (ROSC).

## Supplementary Figure 19. Exploratory meta-analysis of adjusted odds ratios ROSC comparing supraglottic airway (SGA) versus tracheal intubation (TI). CI = Confidence Interval

## Supplementary Figure 20. Leave-one-out sensitivity analyses (Favorable Neurological Outcome). RR: risk ratio; CI: Confidence Interval; PI: Prediction Interval.

## Supplementary Figure 21. Subgroup analysis according to timing of neurological outcomes. CI: Confidence Interval; NR: Not Reported- studies did not report the time point used to define favorable neurological outcomes.

## Supplementary Figure 22. Trial sequential analysis for favorable neurological outcome.

## Supplementary Figure 23. Exploratory meta-analysis of adjusted odds ratios Favorable Neurological Outcome comparing supraglottic airway (SGA) versus tracheal intubation (TI). CI = Confidence Interval
